# Both Maintenance and Avoidance of RNA-Binding Protein Interactions Constrain Coding Sequence Evolution

**DOI:** 10.1093/molbev/msx061

**Published:** 2017-01-30

**Authors:** Rosina Savisaar, Laurence D. Hurst

**Affiliations:** 1 The Milner Centre for Evolution, Department of Biology and Biochemistry, University of Bath, Bath, United Kingdom

**Keywords:** RNA-binding proteins, dual coding, avoidance selection, synonymous sites

## Abstract

While the principal force directing coding sequence (CDS) evolution is selection on protein function, to ensure correct gene expression CDSs must also maintain interactions with RNA-binding proteins (RBPs). Understanding how our genes are shaped by these RNA-level pressures is necessary for diagnostics and for improving transgenes. However, the evolutionary impact of the need to maintain RBP interactions remains unresolved. Are coding sequences constrained by the need to specify RBP binding motifs? If so, what proportion of mutations are affected? Might sequence evolution also be constrained by the need not to specify motifs that might attract unwanted binding, for instance because it would interfere with exon definition? Here, we have scanned human CDSs for motifs that have been experimentally determined to be recognized by RBPs. We observe two sets of motifs—those that are enriched over nucleotide-controlled null and those that are depleted. Importantly, the depleted set is enriched for motifs recognized by non-CDS binding RBPs. Supporting the functional relevance of our observations, we find that motifs that are more enriched are also slower-evolving. The net effect of this selection to preserve is a reduction in the over-all rate of synonymous evolution of 2–3% in both primates and rodents. Stronger motif depletion, on the other hand, is associated with stronger selection against motif gain in evolution. The challenge faced by our CDSs is therefore not only one of attracting the right RBPs but also of avoiding the wrong ones, all while also evolving under selection pressures related to protein structure.

## Introduction

One of the most captivating problems in molecular evolution is that of multiple coding − how the very same DNA sequence can contain several overlapping layers of information. This was once believed to primarily characterize viral genomes, where open reading frames (ORFs) routinely overlap (Barrell et al. 1976; Normark et al. 1983; Belshaw et al. 2007; Chirico et al. 2010). It is understood now, however, that not only are overlapping genes more common in vertebrates than previously believed (Veeramachaneni et al. 2004; Michel et al. 2012), other forms of multiple coding are near-ubiquitous (Itzkovitz et al. 2010; Lin et al. 2011; Shabalina et al. 2013; Pancsa and Tompa 2016). For example, protein-coding regions can overlap with transcription factor binding sites (Stergachis et al. 2013; Birnbaum et al. 2014) (although the functionality of the sites is contested; Xing and He 2015; Agoglia and Fraser 2016), functional RNA secondary structures (Chamary and Hurst 2005; Meyer and Miklos 2005; Pedersen et al. 2006; Smith et al. 2013) and microRNA targets (Lewis et al. 2005; Hurst 2006; Forman et al. 2008; Fang and Rajewski 2011; Hausser et al. 2013; Liu et al. 2015). This means that the evolution of coding sequences (CDSs) is directed not only by selection pressures related to the structure of the protein encoded for but also by the need to preserve such overlapping regulatory information.

Here, we have examined one particular layer of information in CDSs, namely target sites to RNA-binding proteins (RBPs). A constantly changing assortment of RBPs accompanies the (pre-)mRNA transcript throughout its life and coordinates gene expression (Glisovic et al. 2008; Muller-McNicoll and Neugebauer 2013; Singh et al. 2015). Although many of these proteins interact preferentially with untranslated regions (UTRs) or introns (Licatalosi et al. 2008; Xue et al. 2009; Ince-Dunn et al. 2012), others primarily bind CDSs (Grellscheid et al. 2011; Änkö et al. 2012; Ascano et al. 2012). We have sought to quantify to what extent the evolution of CDSs is constrained by the need to preserve or to avoid interactions with RBPs.

To do so, we have studied the enrichment and conservation of particular *k*-mers within CDSs. At least some RBPs recognize and interact with particular (classes of) sequence motifs in the mRNA (Ray et al. 2013; Li et al. 2014). If such an RBP functionally binds within CDSs, then this should lead to the over-representation and excess conservation (compared with null/neutral expectations) of the relevant motifs. On the other hand, it is possible that target sites to other sequence-specific RBPs are avoided in CDSs if interactions between CDSs and those RBPs have deleterious consequences. For example, if an RBP that normally functions by binding introns bound a CDS, it could theoretically interfere with exon definition during pre-mRNA processing or simply constitute a waste of the protein. Such avoidance should manifest itself in the associated motifs being less frequent than expected by chance. The impact on evolutionary rates should be 2-fold: on the one hand, the avoided motifs themselves are expected to be fast-evolving due to pressure to degrade them. On the other hand, those *k*-mers that are a short mutational distance away from an avoided motif should be under selection against substitutions that would give rise to the avoided motif.

Such patterns of enrichment and conservation have been studied extensively for exonic splice enhancers (ESEs). ESEs are short RNA motifs, enriched at exon ends, that promote splicing and are important for the correct identification of the splice sites in a wide range of multicellular organisms (Blencowe 2000; Fairbrother et al. 2004; Wu et al. 2005; Wang and Burge 2008; Warnecke et al. 2008; Cáceres and Hurst 2013). They are under purifying selection (Fairbrother et al. 2004; Carlini and Genut 2006; Parmley et al. 2006, 2007; Ke et al. 2008; Sterne-Weiler et al. 2011; Cáceres and Hurst 2013), leading in human and mouse to an estimated reduction in the over-all rate of evolution at synonymous sites of ∼1.9–4% (Parmley et al. 2006; Cáceres and Hurst 2013). There is evidence that the pressure to conserve ESEs may also have an impact on protein evolution: higher ESE density, as well as higher splice factor binding site density, have been found to correlate with increased protein disorder (Macossay-Castillo et al. 2014; Smithers et al. 2015). Moreover, Parmley et al. (2007) showed that amino acid composition at exon ends, where ESEs are most frequent, is biased towards residues that are encoded for by codons that are frequent in ESEs (for a case study, see Falanga et al. 2014). More generally, there is evidence that the proportion of an mRNA that is within a short distance to a splice site (and therefore likely enriched in splice regulatory information) is one of the main determinants of how fast the corresponding protein evolves (Parmley et al. 2007).

Most ESE function can probably be explained by interactions with RBPs, notably SR proteins (Blencowe 2000; Zhou and Fu 2013). The work discussed above on the evolution of these motifs therefore constitutes a step towards understanding the evolutionary importance of RBP binding more generally. In the present study, we expanded the scope of the analysis from splice enhancement alone to all the functions CDS–RBP interactions may have (see “An Estimate for the Decrease in the Synonymous Rate of Evolution That Is Due to Selection to Preserve Interactions with RNA-Binding Proteins” section of the “Discussion” section for further consideration of the functions of RBPs).

We assembled a large set of *k*-mers that have been demonstrated experimentally to be recognized by various RBPs, and scanned human CDSs for hits. Note that we are concerned strictly with global biases on mRNA sequence evolution and not with predicting individual binding sites, a separate problem that would require a different approach (see “Materials and Methods” section for discussion). We found the motifs to be both more frequent and more conserved than would be expected by chance from their nucleotide composition. We estimate the net effect of the need to preserve them to be a decrease of ca. 2.4% in the over-all rate of evolution at human synonymous sites − an estimate that is in line with those produced previously for ESEs (Parmley et al. 2006; Cáceres and Hurst 2013). This might suggest that ESEs alone capture a large fraction of the selective pressures acting on motifs recognized by RBPs as a whole.

Importantly, the task facing CDSs appears to be not simply to maintain necessary RBP interactions but also to avoid inappropriate ones. Indeed, although the over-all effect is one of motif enrichment, there are also many RBPs whose putative target motifs are depleted compared with nucleotide-controlled null, and appear to be selectively avoided in CDS evolution. It is possible that these represent RBPs whose interactions with CDSs can have deleterious consequences, either because they actively interfere with gene expression or because they divert the protein away from functional binding sites in other transcript regions.

## Results

### Putative RBP Target Motifs Are Nonneutrally Evolving in CDSs, Leading to an Over-All Decrease of ∼2.4% in the Human Rate of Synonymous Evolution

#### Putative RBP Target Motifs Are Enriched over Expected in CDSs

Is the frequency of putative RBP target motifs in CDSs consistent with neutral expectations or are there deviations that would suggest the presence of selection? We retrieved data on the experimentally determined sequence specificities of human RBPs from several databases. This provided us with 114 RBPs, each one associated to a particular set of *k*-mers with *k* ranging from 5 to 12 (from now on these *k*-mers will be referred to as *RBP motifs*; additional file S1, Supplementary Material online). The motifs were pooled across all the sets, resulting in a final list of 1483 unique RBP motifs. The techniques used to determine these motifs vary widely, ranging from nuclear magnetic resonance based approaches (Garcia-Mayoral et al. 2008) to high throughput competition assays, such as RNAcompete (Ray et al. 2013). We next compiled a set of 10,337 full human intron-containing CDSs (concatenations of all the coding regions from the transcript variant with the longest CDS). To alleviate problems of statistical nonindependence, the CDSs were clustered into families of paralogs (additional file S2, Supplementary Material online). In the analyses described below, statistics were either averaged within families or only a single randomly picked gene was considered from each, resulting in 5,845 independent data points for each estimate (see “Materials and Methods” section for further details).

We then scanned the CDSs for RBP motifs and calculated the motif density, that is to say, the fraction of the bases in a given CDS that overlapped with any of the motifs. The median density was ≈0.573 (supplementary spread sheet 1 in additional file S4, Supplementary Material online), meaning that over half of the sequence in a typical human intron-containing CDS overlaps with one or more RBP motifs. Does this deviate from the density that would be expected by chance for a set of motifs of this size and of this base content? We generated 1,000 sets of simulant motifs of the same size and roughly the same dinucleotide composition as the set of RBP motifs. We determined the density of the simulant sets in our sequences and observed that none of them had a median density as high as that observed with real RBP motifs. RBP motifs are therefore enriched in CDSs with a *P* value of ≈0.001 ($P=\frac{n+1}{m+1}$, where *n* is the number of simulant sets that present a median density as great as or greater than that observed with the real motif set and *m* is the total number of simulant sets). This is an indication that there could indeed be selection to preserve these motifs.

In order to quantify this enrichment, we can calculate a normalized density value for each gene ($ND=\frac{truedensity–meanofsimulateddensities}{meanofsimulateddensities}$). ND is a measure of enrichment over the nucleotide-controlled null. An ND value of 0 signifies that the motifs are about as frequent as would be expected by chance given their nucleotide composition, whereas an ND of 1 means that they are twice as frequent as expected and an ND of −0.5 that they are half as frequent. For RBP motifs, we recover a median ND value of ≈ 0.115.

#### RBP Motifs Are under Purifying Selection

If the motif enrichment reported above truly reflects the functionality of (a subset of) the *k*-mers rather than, say, a methodological bias in the simulations, then in addition to being enriched, the motifs should also be slower-evolving than expected from their nucleotide composition. To test this prediction, we aligned the gene regions overlapping the motifs to the homologous regions in the macaque (*Macaca mulatta*) genome and calculated the rate of evolution at synonymous sites (*d_S_*). We then applied the same procedure to each of the 1,000 simulated versions of the RBP motifs set. This generated a distribution of simulant *d_S_* estimates, from which we calculated an empirical conservation *P* value ($P=\frac{n+1}{m+1}$, where *n* is the number of simulant sets that present a *d_S_* as low as or lower than that observed with the real motif set and *m* is the total number of simulant sets) and a normalized *d_S_* estimate ($normalizedd_{S}=\frac{trued_{S}–meanofsimulatedd_{S}}{meanofsimulatedd_{S}}$). RBP motifs show a significant reduction in *d_S_* (raw *d_S_* ≈ 0.064; normalized *d_S_* ≈ −0.041; *P* ≈ 0.003). This suggests that CDSs are indeed under selection to preserve RBP motifs, underlining their functionality.

In order to further verify this result using a different method, we compared evolutionary rates at 4-fold degenerate sites that overlapped RBP motifs to rates at those that did not, performing the analysis separately for each dinucleotide (see supplementary text 1 and figure S1 in additional file S5, Supplementary Material online, for details). Although the effects recovered were weaker than those obtained in the *d_S_* analysis reported above, the majority of dinucleotides do evolve more slowly within RBP motifs than elsewhere (*χ*^2 ^≈^ ^4, *P* < 0.05 from *χ*^2^ test; *P* ≈ 0.017 from a paired one-tailed Wilcoxon signed rank test comparing the rates obtained for each dinucleotide in motifs and nonmotifs). It appears therefore that our results cannot simply be due to a bias in the normalization procedure that would cause a few fast-evolving dinucleotides (such as *CG*) to be over-represented in simulants when compared with the true motifs.

#### RBP Motif Enrichment Is Stronger in Genes That Are Expressed More Tissue-Specifically

The hypothesis of RBP motif functionality possibly makes a further prediction, namely that the motifs should be enriched more in genes that are more highly expressed or expressed in a greater number of tissues. This is because various errors made during gene expression should have greater fitness consequences if the transcript is more abundant, assuming that all else is equal. Selection on regulatory signals that help ensure correct gene expression should therefore be stronger, leading to higher enrichment.

We obtained FANTOM5 expression data (Fantom Consortium et al. 2014) for the genes in our dataset. For each gene, we calculated the following expression parameters: expression breadth (fraction of tissues where the gene is expressed), median expression, maximum expression, and median expression in tissues where the gene is expressed (supplementary spread sheet 18 in additional file S4, Supplementary Material online). After Bonferroni correction, we find that ND indeed correlates significantly with three of these variables (table 1). However, contrary to our expectations, the sign of the correlation is negative rather than positive. In addition, the relevant parameter seems to be the number of tissues in which the gene is expressed more so than transcript abundance in any given tissue. In other words, it appears that the more tissue-specific a gene’s expression pattern, the more RBP motifs are enriched. This might reflect greater levels of regulation in more narrowly expressed genes. This tendency must be stronger than any increased purifying selection on genes with greater expression breadth.

**Table 1:** Spearman Correlation between Normalized Density (ND) and Various Expression Parameters, Determined Based on FANTOM5 Data.

	Expression Breadth (fraction of tissues where gene is expressed^a^)	Maximum Expression	Median Expression	Median Expression in Tissues Where the Gene Is Expressed
*ρ*	≈−0.151	≈−0.035	≈−0.157	≈−0.016
*P*^b^	≈9.576×10^−30^ (≈3.830×10^−29^)	≈0.010 (≈0.038)	≈3.071×10^−32^ (≈1.228×10^−31^)	≈0.280 (1.000)

a A gene is considered to be expressed in a given tissue if more than five tags per million map to the promoter region (see “Materials and Methods” section for further details).

b The parentheses contain the Bonferroni-corrected *P* value.

We were concerned that the negative correlation between ND and expression parameters could be reflecting properties of simulant motifs rather than of the true RBP motifs. Namely, the formula for calculating ND ($ND=\frac{truedensity–meanofsimulateddensities}{meanofsimulateddensities}$) requires one to divide by the mean of simulated densities. If simulated density correlated positively with expression breadth, this could lead to a negative correlation between ND and expression breadth without there being any relationship between true motif density and expression. We therefore repeated the analysis using *Z*-scores rather than ND ($Z=\frac{truedensity–meanofsimulateddensities}{standarddeviationofsimulateddensities}$). *Z*-scores should be more robust to fluctuations in the simulated mean, as this parameter does not appear in the denominator. It is therefore reassuring that we observed a negative correlation between *Z* and expression breadth (*ρ *≈ −0.156, *P* < 2.2×10 ^−^ ^16^; Spearman rank correlation). In addition, raw motif density also correlates negatively and significantly with expression breadth (*ρ *≈ −0.123, *P* < 2.2×10 ^−^ ^16^; Spearman rank correlation), demonstrating that the effect we observe for ND cannot be explained solely by patterns of simulated density.

To conclude, although the sign of the correlation is different from what was hypothesized, the fact that RBP motif enrichment correlates significantly with expression parameters adds further support to the claim that these motifs are functional in CDS.

#### The Need to Preserve RBP Motifs Leads to an Over-All Reduction of ∼2 – 3% in Primate and Rodent d_s_

It has been estimated (Parmley et al. 2006; Cáceres and Hurst 2013) that the need to preserve ESEs causes a reduction of about 1.9–4% in the over-all rate of evolution at synonymous sites (*d_S_*). What would be the analogous estimate for RBP motifs? To find out, one can multiply normalized *d_S_* by ≈0.573, that is to say, the fraction of the sequence in the median human CDS that is made up of RBP motifs. This provides us with an estimate for the over-all reduction in the *d_S_* of the median gene that can be attributed to the pressure to preserve RBP motifs. This statistic turns out to be ≈−0.024. It therefore appears that the need to preserve RBP motifs indeed places a weak but detectable constraint on sequence evolution within human protein-coding regions. The magnitude of the effect we report for RBP motifs in CDSs is in line with previous estimates obtained for ESEs. However, not all RBP motif-related constraint seems to be splice-associated: the net decrease in *d_S_* is similar between intron-containing and intronless sequences (supplementary text 2 in additional file S5, supplementary spread sheet 7 in additional file S4, and additional file S3, Supplementary Material online), suggesting that splicing-independent factors are important in directing RBP motif evolution.

We next asked whether our results concerning selection on RBP motifs in CDSs could be confirmed in another system. We repeated the analysis on 15,631 mouse (*Mus musculus*) CDSs, using motifs derived for mouse RBPs (additional file S1; supplementary spread sheet 10 in additional file S4, Supplementary Material online). We employed the rat (*Rattus norvegicus*) genome for estimating conservation. We recovered a lower median motif density than in human (≈0.339 and ≈0.573, respectively). However, this is likely simply because the set of motifs was smaller in mouse (736 motifs compared with the 1,483 in human). The extent of enrichment (ND ≈ 0.128; *P* ≈ 0.010) was similar to that obtained in human. Excess conservation was slightly more pronounced (raw *d_S_* ≈ 0.165; normalized *d_S_* ≈ −0.063; *P* ≈ 0.010), leading to an estimate of ≈2.1% for the over-all reduction in *d_S_* that would be due to the need to preserve RBP motifs. Data from mouse therefore also provides evidence for purifying selection on RBP motifs, and leads to similar conclusions with regards to the magnitude of this constraint.

#### RBP Motif-Related Constraint Is As Strong in CDSs As It Is in Introns and UTRs

We have provided evidence that RBP motifs are under selection in CDSs. However, is the over-all evolutionary impact of this selection substantially weaker in CDSs than in the noncoding regions of protein-coding genes? This might be expected as the latter regions are not under the additional constraint of specifying protein structure. They could therefore be particularly prone to the accumulation of regulatory signals, such as RBP binding sites. We analysed RBP motif density and conservation in 5′-UTRs, 3′-UTRs, full introns and exon proximal intronic regions (the 100-bp immediately upstream or downstream from an exon; supplementary spread sheets 13–17 in additional file S4, Supplementary Material online). We found evidence for RBP motif conservation in all compartments and in all bar the intronic sequence from the downstream flanks of exons the effect was significant (table 2).

**Table 2:** Motif Density and Conservation Parameters for Various Genic Regions.

	CDSs	5′-UTRs	3′-UTRs	Introns	Upstream Intronic^a^	Downstream Intronic^a^
Median motif density	≈0.573	≈0.537	≈0.573	≈0.578	≈0.580	≈0.560
Median ND^b^	≈0.115	≈0.145	≈0.103	≈0.129	≈0.167	≈0.130
Enrichment *p*^c^	≈0.001	≈0.010	≈0.010	≈0.010	≈0.010	≈0.010
*d_NC_*^d^ or *d_S_*^e^	≈0.064	≈0.052	≈0.043	≈0.055	≈0.051	≈0.054
Normalized *d_NC_*^d^ or normalized *d_S_*^e^	≈−0.041	≈−0.019	≈−0.026	≈−0.034	≈−0.035	≈−0.017
Conservation *p*^c^	≈0.003	≈0.030	≈0.040	≈0.010	≈0.030	≈0.149
Global reduction^f^	≈−2.4%	≈−1.0%	≈−1.5%	≈−2.0%	≈−2.0%	≈−0.9%

a Upstream/downstream intronic regions correspond to 100 bp slices immediately upstream/downstream from an exon.

b Normalized density.

c One-tailed *P* derived from an empirical distribution of simulant statistics. 1,000 simulants were used for CDSs and 100 in the other cases.

d Rate of evolution at noncoding sites. Used for all sequence regions except for CDSs.

e Rate of evolution at synonymous sites. Used for CDSs.

f The global reduction is the product of the motif density and the conservation statistic (multiplied by 100). It is an estimate for the extent to which the (synonymous) substitution rate is decreased in the relevant region because of selection to preserve RBP motifs. Note that in the table, the density and the normalized conservation estimates have been rounded to the third decimal, whereas exact values were used when calculating the global reduction.

Contrary to our expectations, the over-all constraint (the product of the motif density and the nucleotide-normalized conservation estimate) was stronger in CDSs than in any of the noncoding regions (table 2). This could be reflecting the fact that synonymous sites are not subject to selective pressures related to amino acid sequence. The selection acting on noncoding signals in CDSs could therefore be disproportionately concentrated at synonymous sites, leading to a strong effect at the level of *d_S_*. However, any such reasoning should be taken with a grain of salt as the conservation statistics were obtained slightly differently for CDSs and for the other sequence regions (using PAML *codeml* for CDSs and PAML *baseml* for the noncoding sequence). We therefore merely note that we find no evidence for unusually weak RBP motif-related constraint in CDSs, and refrain from drawing conclusion from more fine-scale comparisons.

In conclusion, we have attempted to quantify the extent to which excess conservation at RBP motifs leads to a global decrease in *d_S_*. We have found this figure to be about 2.4% − approximately the same level of constraint as can be observed in the noncoding regions of protein-coding genes. We emphasize that the figures we provide are to be taken as rough estimates only, as they are sensitive to the number of motifs defined as RBP motifs and to the procedure used for calculating the neutral expectation. Note also that our approach does not discriminate between strong selection acting on a few of the motifs in our set and weak selection acting on many. In the sections to follow, we will attempt to clarify this issue.

### Nucleotide-Controlled Density Varies Greatly among Motifs Putatively Recognized by Different RBPs, with Depletion No Less Frequent than Enrichment

#### When RBP Motifs Are Grouped Based on the Cognate RBP, the Enrichment P Values of the Resulting Motif Sets Distribute Bimodally

We determined above that RBP motifs were both more frequent and more conserved in CDSs than expected from their nucleotide composition, leading to a slight decrease in over-all *d_S_*. It remains unclear, however, what the contributions of the motifs putatively recognized by different RBPs are to this result. Are more or less all RBP motifs enriched over expected or is the over-all enrichment largely driven by a subset of the motifs? Could some RBP motifs be not enriched but depleted instead? For instance, an intronic splice regulator binding within an exon could hypothetically interfere with exon recognition and so the presence of the cognate motifs within exons might be deleterious.

We repeated the analysis of motif density but instead of pooling the motifs, we considered the *k*-mers associated to each RBP separately. From here on, we will use the phrase *motif set* to refer to the motifs putatively recognized by a particular RBP. In total, there are therefore 114 motif sets, each corresponding to one RBP (see supplementary spread sheet 19 in additional file S4, Supplementary Material online, for the sizes of the motif sets). As above for the pooled analysis, we generated 1,000 approximately dinucleotide-matched simulated versions of each motif set so that we could calculate ND and an enrichment *P* for the motifs putatively recognized by each RBP (supplementary spread sheet 2 in additional file S4, Supplementary Material online).

**Fig. 1.**
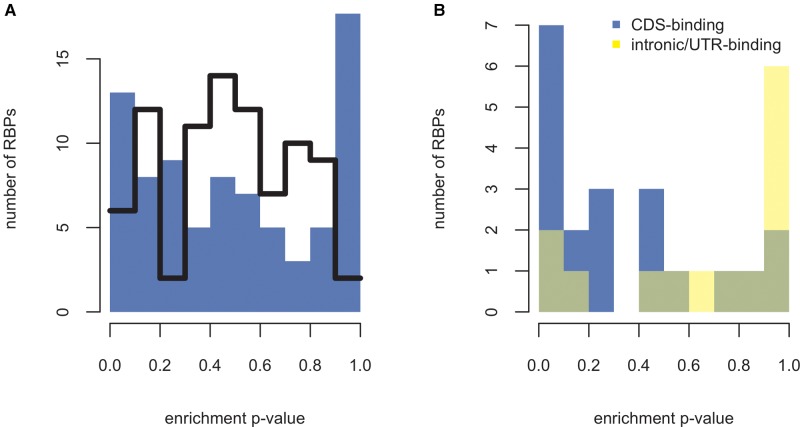
(*A*) Each data point corresponds to the probability that a given motif set (recognized by a particular RBP) would be found at its current density (or higher) by chance given the underlying dinucleotide composition. The black line traces the distribution of enrichment *P* values obtained in the same sequences for size-matched sets of random *k*-mers. Note that RBP motifs display a peak at either extreme of the distribution whereas the random motifs do not. In other words, RBP motifs show a disproportionate tendency to occur at a density that deviates from neutral expectations. Importantly, this can mean both enrichment (*P* value approaching 0) and depletion (*P* value approaching 1). (*B*) As *A*, except that only RBPs for which we found crosslinking and immunoprecipitation studies on binding preferences are shown. Motif sets associated to CDS-binding RBPs (blue) have a peak near 0 (enrichment), whereas the other sets (yellow) have a peak near 1 (depletion).

Some motif sets were very rare, leading to concerns over the reliability of estimating ND and other parameters in such cases. Because of this issue, we removed motif sets where hits to neither the true motifs nor the simulant sets reached a pre-defined density threshold (see “Materials and Methods” section). After this filtering step, 81 motif sets remained (additional file S1, Supplementary Material online), containing a total of 1,213 unique motifs. The enrichment *P* values obtained for most of them were nonsignificant. However, there was a peak at either extreme (near 0 and near 1) when they were plotted out as a histogram (fig. 1*A*), leading to a significantly nonunimodal distribution (*D* ≈ 0.069; *P* ≈ 0.005; Hartigans’ dip test).

In other words, a large proportion of the motifs fall into one of two classes: a (near-) significantly enriched class and a (near-)significantly depleted class. The over-all enrichment over expected that is obtained when all the motifs are pooled is therefore the average of many competing trends: the motifs putatively recognized by some RBPs are enriched, whereas others distribute at random frequencies or are altogether depleted.

#### The Bimodal Distribution of Enrichment P Values Is Specific to RBP Motifs

Is this bimodal distribution of *P* values specific to RBP motifs or could it be an artefact of our method for estimating *k*-mer enrichment? In the latter case, a similar distribution of *P* values should also occur with motifs that are not expected to be biologically meaningful. We therefore replaced each motif within each motif set with a random *k*-mer of the same length and repeated the density analysis with these random motifs. We then generated 1,000 sets of approximately dinucleotide-matched simulant motifs for each random motif set in order to calculate the enrichment *P* values, identically to the analysis performed above for RBP motifs.

Unlike the RBP motifs, the random motifs showed no tendency for extreme *P* values (black line in A; supplementary spread sheet 3 in additional file S4, Supplementary Material online). To formally confirm this visual observation, we classed the *P* values into two groups: below 0.1 or above 0.9, and between 0.1 and 0.9 (included). We then counted the number of *P* values in either group and found the proportion to be significantly different for the RBP motifs and for random *k*-mers (*χ*^2 ^=^ ^75.593, *P* < 0.001). In order to test the significance of the depletion effect specifically, we also compared the proportion of *P* values above 0.9 to those below or equal to 0.9 for RBP motifs and for random *k*-mers. This difference was also significant (*χ*^2 ^=^ ^132.819, *P* < 0.001). The bimodal distribution of enrichment *P* values is therefore unlikely to result from methodological biases. We also considered the possibility that differences in stop codon content between the motifs and their simulants could be contributing to the depletion observed. The details of this analysis can be found in supplementary text 3 in additional file S5, and supplementary figures S2 and S3, also in additional file S5, Supplementary Material online. Briefly, we found that although this factor might play some role in determining ND, it does not seem to explain the over-all pattern.

In conclusion, the tendency for extreme enrichment *P* values exhibited by RBP motif sets is probably not due to methodological factors, as control motifs not thought to be biologically meaningful do not display this pattern. It is therefore likely that it is a reflection of the functionality of at least some of the motif sets.

### The Variation in Enrichment between Different Sets of RBP Motifs Likely Reflects Functional Differences

#### Motif Sets That Are More Strongly Enriched Also Tend to Be More Conserved

**Fig. 2.**
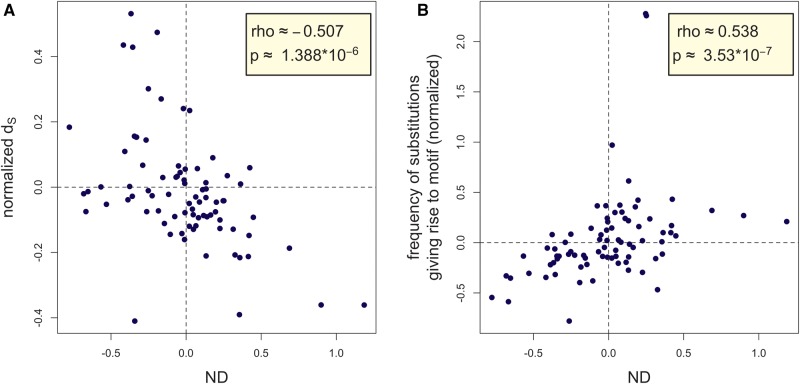
(*A*) Correlation between a motif set’s normalized density (ND) and its nucleotide-normalized *d_S_* from alignment to macaque. Motif sets that are more strongly enriched are also more conserved, controlling for nucleotide composition. The dashed lines intersect the plot at the points where expected and observed frequencies would be equal. (*B*) Correlation between ND and the nucleotide-normalized propensity to gain the motifs over evolution (measured by determining how frequently macaque sites that are orthologous to human 4-fold degenerate sites that are a single base substitution away from the motif in human contain the base that would give rise to the motif in human). Note that because our analysis did not make use of an outgroup, we cannot know on which branch the substitution occurred in cases where the human and macaque sequence differ. See caption to subplot A for interpretation of the dashed lines.

We have seen above that the extent of enrichment varies between sets of motifs putatively recognized by distinct RBPs. If this variation reflects differences in the functional importance of the motifs, then it should correlate with evolutionary rate: those motif sets that are more enriched should also be more conserved. To test this prediction, we calculated *d_S_*, normalized *d_S_* and a conservation *P* value separately for each motif set (supplementary spread sheet 4 in additional file S4, Supplementary Material online). As predicted under a functional hypothesis, we recovered a significant correlation between a motif set’s ND and its normalized *d_S_* (*ρ*** **≈ −0.507; *P* ≈ 1.388×10 ^−^ ^6^; Spearman rank correlation; fig. 2*A*; see supplementary fig. S4 in additional file S5, Supplementary Material online, for qualitatively similar results obtained using enrichment *Z*-scores instead of ND, which controls for differences in the variance of the simulated density values; see supplementary fig. S5 in additional file S5, Supplementary Material online, for a version of fig. 2 where each data point is labelled according to the associated RBP). Similarly, there is a significant positive correlation between enrichment *P* values and conservation *P* values (*ρ*** **≈ 0.503, *P* ≈ 1.75×10 ^−^ ^6^; Spearman rank correlation). The variation in the extent of enrichment, therefore, indeed likely results from functional differences between sets.

We repeated this analysis also for intronless CDSs and recovered similar patterns to those observed in intron-containing ones, once again underscoring the importance of processes other than splicing for determining RBP motif usage and evolution (supplementary text 2 in additional file S5, and supplementary spread sheets 8 and 9 in additional file S4, Supplementary Material online). We also performed the analysis using mouse CDSs and mouse RBPs (supplementary spread sheets 11 and 12 in additional file S4; additional file S2, Supplementary Material online). Like in human, we obtained a significant negative correlation between ND and normalized *d_S_* (*ρ*** **≈ −0.312; *P* ≈ 0.005; Spearman rank correlation), and a significant positive correlation between enrichment *P* values and conservation *P* values (*ρ*** **≈ 0.352; *P* ≈ 0.001; Spearman rank correlation).

It could be pointed out that there is a significant correlation between the ND and the raw density of motif sets (*ρ*** **≈ 0.292, *P* ≈ 0.008; Spearman rank correlation), and that the reliability of estimated *d_S_* values is expected to depend on the amount of information available, which in its turn depends on the raw density. Therefore, the correlation between ND and normalized *d_S_* could be due to less noisy estimation of normalized *d_S_* in motif sets with greater ND. This is worrying because raw density is partially determined by methodological factors, such as the number of motifs in the set (*ρ*** **≈ 0.674, *P* ≈ 5.515×10 ^−^ ^12^; Spearman rank correlation between motif number and raw density) and the length of the motifs (*ρ*** **≈ −0.323, *P* ≈ 0.003; Spearman rank correlation between median motif length and raw density). However, this alternative explanation predicts that in addition to the negative correlation between ND and normalized *d_S_*, there should also be one between raw density and normalized *d_S_*. This prediction is incorrect: there is no significant correlation between the raw density of a motif set and its normalized *d_S_* (*ρ*** **≈ 0.007, *P* ≈ 0.949; Spearman rank correlation). This confound is therefore unlikely to explain our results. We also note that several of the motif sets that present particularly extreme values for both ND and for normalized *d_S_* are composed of very few motifs (see, for instance, CUGBP, Elav-Like Family Member 1 [CELF1] and Sterile Alpha Motif Domain Containing 4A [SAMD4A] in supplementary fig. S5 in additional file S5, Supplementary Material online) and might therefore give rise to less reliable estimation of normalized *d_S_*. Could our results be due to the presence of noisy outliers? This does not seem to be the case: we repeated the analysis after having removed all motif sets with fewer than five motifs and the significant correlation between ND and normalized *d_S_* remained (*ρ*** **≈ −0.520, *P* ≈ 5.773×10 ^−^ ^4^; Spearman rank correlation).

It therefore appears likely that the motif sets that show the strongest enrichment are those recognized by RBPs whose interactions with CDSs are the most important to maintain. Do the associated RBPs also show preferential binding in CDSs in experimental studies? We annotated the RBPs as either *CDS-binding*, *non-CDS-binding* or *unknown* based on published high-throughput crosslinking and immunoprecipitation studies (CLIP-Seq) (Licatalosi et al. 2008; Xue et al. 2009; Hafner et al. 2010; Konig et al. 2011; Van Nostrand et al. 2016) (see supplementary spread sheet 5 in additional file S4, Supplementary Material online, for references to data sources). The motif sets that were associated with CDS-binding RBPs indeed had greater raw density (*P* ≈ 0.016; one-tailed Mann–Whitney *U*-test), greater ND (*P* ≈ 0.006; one-tailed Mann–Whitney *U*-test) and lower enrichment *P* values (*P* ≈ 0.009; one-tailed Mann–Whitney *U*-test; fig. 1*B*) than those annotated as non-CDS-binding. This concordance with experimental data both lends credence to the motif to RBP mapping and provides further support for the functional relevance of our observations.

In summary, the motif sets that are more strongly enriched in CDSs also tend to be slower-evolving, suggesting that they represent a subset of RBP motifs whose presence in CDSs has particular functional importance.

#### The Depletion of Certain Motif Sets Is Likely Due to Purifying Selection to Avoid Them

We noted above that despite the over-all enrichment of RBP motifs over nucleotide-controlled null in CDSs, many of the motif sets associated to individual RBPs were depleted instead. As this depletion is not observed for random *k*-mers (black line in fig. 1*A*), it most likely reflects selection to avoid motifs recognized by RBPs whose interactions with CDSs can be deleterious, either because they constitute a waste of the protein on inappropriate binding or because they interfere with gene expression. The latter type of scenario is easy to imagine in the case of splicing: an exon is partially defined by the factors that bind to it and so a change in the complement of binding partners could hypothetically interfere with exon recognition.

The implications of this avoidance for CDS evolution are likely 2-fold. Firstly, one expects purifying selection against the avoided motifs, resulting in a general constraint on the sequence space available in CDS evolution. A read-out of this effect would be a rarity of substitutions that give rise to an avoided motif. Secondly, when the avoided motifs do occur, there should be positive selection to degrade them. They should therefore be faster-evolving than expected from their nucleotide composition. The magnitude of the second selection pressure will depend on the efficiency of the first: if the purifying selection against the avoided motifs is sufficiently strong, then they might almost never go to fixation in a context where their presence is deleterious. For instance, it could be that the majority of the hits observed for such motifs are in locations where the local mRNA secondary structure prevents the RBP from accessing the site and so these motifs, although present in the sequence, would very infrequently actually interact with the RBP. In this case, no positive selection to lose the motifs is expected and the avoided motifs should instead be neutrally evolving. It is also possible that certain RBP-CDS interactions, although deleterious in most cases, can be adaptive when they occur at very specific locations. In this latter scenario, the avoided motifs would be rare but under purifying selection when present.

In order to determine whether there was any evidence for selection to degrade certain motifs, we pooled the motifs from those sets whose enrichment *P* value was above 0.9 in intron-containing CDSs (the strongest candidates for being avoided; from here on, we will refer to these motifs as the *depleted group*) and calculated their density and rate of evolution in intron-containing CDSs. This resulted in a set of 432 motifs with a median density of ≈0.069, a median ND of ≈−0.130 and an enrichment *P* value of 1 (i.e., significant depletion). There is no evidence for positive selection on the motifs: rather, they are evolving at roughly the rate that would be expected by chance from their nucleotide composition (raw *d_S_* ≈ 0.068; normalized *d_S_* ≈ 0.011; conservation *P* ≈ 0.600). This might suggest that purifying selection to avoid the motifs is sufficiently efficient to mostly prevent them from going to fixation at locations where their presence is deleterious. It is also possible, however, that because of the rarity of the depleted group motifs, we simply lack the power to pick up on any positive selection that is occurring. Moreover, if the avoidance only concerns certain gene regions, this might dilute any signal of positive selection further. For instance, the avoidance might be stronger in the outer regions of exons (the exon flanks) than at the very centre, as the flanks appear to be more crucial for splice regulation. This is evidenced by their enrichment in both splice-altering (Woolfe et al. 2010) and pathogenic (Wu and Hurst 2016) single-nucleotide polymorphisms.

To test this hypothesis, we extracted 69 base pairs from the extreme 5′ end, the extreme 3′ end and the very centre of 4563 human internal coding exons and calculated the *d_S_* of the depleted group. In the 5′ flank, depleted group motifs are indeed evolving faster than expected from their base composition but this effect is nonsignificant (5′ flanks: raw *d_S_* ≈ 0.073; normalized *d_S_* ≈ 0.116; conservation *P* ≈ 0.915). In exon cores and 3′ flanks, however, the same motifs are evolving at chance rates (cores: raw *d_S_* ≈ 0.068; normalized *d_S_* ≈ 0.024; conservation *P* ≈ 0.635; 3′ flanks: raw *d_S_* ≈ 0.072; normalized *d_S_* ≈ 0.045; conservation *P* ≈ 0.727). Given the nonsignificance of the effects, it appears that even when considering the different exonic sub-regions separately, there is little evidence for increased rates of evolution in regions overlapping depleted group motifs.

We next sought to directly test for purifying selection against the depleted group. We determined all 4-fold degenerate sites in our set of intron-containing CDSs such that a single base substitution at the site would give rise to one of these motifs. We then aligned the CDSs to macaque orthologs and found that at ≈1.4% of such sites, the base that would create a depleted group motif were it used at that position in human was indeed present at the orthologous site in the macaque sequence (the site counts have been weighted based on site degeneracy − see “Materials and Methods” section). We repeated the same analysis on 1000 sets of dinucleotide-matched simulant motifs and found the corresponding percentage to be ≈1.6% on average. This difference is slight but significant (one-tailed empirical *P* ≈ 0.009 from the distribution of simulant values). This is evidence for selection against substitutions that would give rise to a depleted group motif.

Another way to test for purifying selection against certain RBP motifs is to consider the variation among motif sets. If motif depletion is largely driven by purifying selection to avoid, it is expected that the more a motif set is depleted, the more motif gain is selected against over evolution. To test this hypothesis, we repeated the analysis of sites that are a single substitution removed from a motif but this time separately for the individual RBP motif sets (supplementary spread sheet 6 in additional file S4, Supplementary Material online). For each motif set, we calculated the fraction of one-removed sites where the base that would give rise to one of the motifs in the set in human was present in macaque. We then normalized this statistic by subtracting from this value the mean fraction observed for simulated sets and dividing the difference by the simulated mean. We then calculated the correlation between these normalized fractions and ND. As predicted, this correlation was significantly positive (*ρ*** **≈ 0.538, *P* ≈ 3.530×10 ^−^ ^7^; Spearman rank correlation; fig. 2*B*). Analysis of individual motif sets therefore also provides evidence that the depletion of certain motif sets is due to purifying selection to avoid them.

The fact that CDSs co-exist in the cell with RBPs therefore has the effect of carving out a sub-region of sequence space within which CDSs preferentially evolve. Deviating from these constraints may not only lead to the loss of necessary CDS-RBP interactions but might also provoke inappropriate ones.

## Discussion

### An Estimate for the Decrease in the Synonymous Rate of Evolution That Is Due to Selection to Preserve Interactions with RNA-Binding Proteins

In this study, we have sought not simply to test whether the need to preserve RBP binding constrains CDS evolution but also to quantify the evolutionary impact of any such dual coding. We estimate that the need to conserve motifs putatively recognized by RBPs leads to a decrease of ca. 2 − 3% in the over-all rate of evolution at synonymous sites in both primates and rodents compared with a nucleotide controlled null. This reduction in evolutionary rate, however, is not distributed uniformly across the RBP motifs, appearing to be driven by a subset of the motifs that are particularly enriched and conserved, while others occur at chance frequencies or are altogether depleted. Note also that the very low figure that we provide for the over-all decrease in evolutionary rates is likely an underestimate because the nucleotide-controlled null has been intentionally designed to be conservative. It is possible that some of the control sites overlap with functional RBP targets and are therefore conserved, leading to an overly low expected rate of evolution.

The estimate that we have produced for RBP motifs is comparable to the 1.9−4% range that can be deduced from similar analyses on exonic splice enhancers (ESEs) (Parmley et al. 2006; Cáceres and Hurst 2013). This might indicate that ESEs alone capture a large fraction of the selective pressures acting on putative RBP target motifs more generally. This should not be taken to imply that all RBP-related constraint is due to the need to ensure correct splicing: we found both the over-all level of constraint, as well as the enrichment and conservation patterns of the individual sets of motifs putatively recognized by particular RBPs, to be remarkably similar between intron-containing and intronless sequences (supplementary text 2 in additional file S5, Supplementary Material online). This suggests that splicing-independent factors may be surprisingly important in shaping the RBP motif content of CDSs. This result concords with previous findings that ESEs are both enriched and conserved (compared with nucleotide-controlled null) also in genes that do not undergo splicing, indicating that they too might be relevant to processes other than splicing (Pozzoli et al. 2004; Savisaar and Hurst 2016).

Our data do not inform us on which particular splicing-independent functions might be the most relevant in directing RBP motif evolution. However, it is well established that RBPs that bind the CDS can indeed have such roles. For instance, the serine-arginine rich splice factor 1 (SRSF1) is crucial for maintaining genome stability (Li and Manley 2005; Tuduri et al. 2009), whilst the serine-arginine rich splice factors 3 and 7 (SRSF3 and SRSF7) have been shown to act as adapters in the nucleo-cytoplasmic transport of the intronless *H2a* mRNA (Huang and Steitz 2001; Huang et al. 2003). Other RBPs, such as fragile X mental retardation 1 (FMR1) (Kao et al. 2010) and Heterogeneous Nuclear Ribonucleoprotein A2 (HNRNPA2) (Shan et al. 2003), are involved in the trafficking of mRNAs within neurons. RBPs that bind in the CDS can also function in translation. This includes roles as both positive (Sanford et al. 2004; Peng et al. 2011) and negative (Darnell et al. 2011) regulators of translation, as well as in the regulation of alternative translation initiation site usage (Bonnal et al. 2005). As a final example, insulin like growth factor 2 mRNA binding protein 1 (IGF2BP1) has been found to stabilize some of its mRNA targets (Noubissi et al. 2006). Several of the RBPs alluded to in this paragraph or in the cited literature are indeed associated to motif sets that have positive normalized density (i.e., are enriched over expected) in intronless CDSs. However, because our method inherently comes with a certain amount of uncertainty with regards to the motif to RBP mapping, we prefer not to draw inferences with regards to the importance of any individual RBPs (see “Materials and Methods” section).

### Evidence That Coding Sequence Evolution Is Constrained by the Need to Prevent Inappropriate Interactions with RBPs

A novel result of this study is the finding that coding regions appear to be under selection to avoid certain RBP motifs. This is supported by evidence for selection against substitutions that would generate such a motif. We also predicted that when the presumed avoided motifs do occur, they would be evolving faster than random expectations, reflecting selection for degradation. We found no such evidence. This may suggest that the purifying selection to avoid the motifs is sufficiently efficient to prevent their fixation in locations where they might have a deleterious effect. Given the rarity of these motifs, however, it is also possible that we simply lack power to detect any increase in evolutionary rates.

This pattern of conserving certain regulatory sequences, yet selectively avoiding others, is likely not specific to RBP motifs but is rather a general feature of genome evolution. Indeed, there is evidence that the 3′-UTRs of genes that are co-expressed with a microRNA are depleted in target sites to that microRNA, most likely to prevent inappropriate down-regulation (Bartel and Chen 2004; Farh et al. 2005; Stark et al. 2005; Chen and Rajewsky 2006), although see Iwama et al. (2007). Other examples of such avoidance selection include selection against spurious transcription factor binding sites in prokaryotes (Hahn et al. 2003) and in yeast (Babbitt 2010), as well as against mononucleotide runs within coding regions in various organisms, potentially to decrease the probability of transcriptional or translational error (Ackermann and Chao 2006; Gu et al. 2010; Itzkovitz et al. 2010). To our knowledge, the present work is the first large-scale study to consider selection to avoid RBP motifs.

Importantly, our results suggest that multiple coding between regulatory and protein structure information is not just about increased purifying selection at the locations where overlapping regulatory signals occur. It also places a more large-scale bias upon the sequence space available in coding region evolution. Not only are regions where necessary regulatory elements appear constrained not to lose them, all coding sequence is expected to be under some level of evolutionary constraint so as not to gain inappropriate signals. The latter constraint is likely to be weaker: given a functional motif, a large fraction of the possible mutations are expected to disrupt it, whereas a much more limited number of mutations would turn a nonmotif into an (avoided) motif.

Finally, we would like to emphasize that our categorization of RBP motifs as preferred or avoided (or neither) is necessarily a gross simplification. Many relevant factors, which might help refine our crude approximations, have not been taken into account. For instance, we have not attempted to predict the mRNA secondary structure around motif hits. This could be relevant, as certain motifs may be preferred/avoided only when the site is accessible. Another important variable that is not considered is that of the context in which the motif hits appear. This includes both the sequence context—the other *k*-mers occurring in the vicinity—and the gene anatomic context, for instance, whether the site is located at an exon end or in the exon core. Some of the motif sets that currently appear to distribute and evolve according to chance expectations might turn out to show evidence of selection once such factors have been accounted for. However, analyses of this type have a great propensity to produce spurious patterns and so they should only be performed with explicit, well-motivated hypotheses in mind.

### Future Directions

Our results indicate that although the need to preserve RBP interactions has a detectable and significant impact on CDS evolution, the effect is slight (though, as touched upon in “An Estimate for the Decrease in the Synonymous Rate of Evolution That Is Due to Selection to Preserve Interactions with RNA-Binding Proteins” section of the “Discussion” section, the figures that we provide are likely underestimates). Studies on ESEs have reached similar conclusions (Parmley et al. 2006; Cáceres and Hurst 2013). However, these results appear, at first sight, to contradict a separate line of work where researchers have experimentally introduced large numbers of mutations into exons to determine the effect on splicing (Pagani et al. 2003, 2005; Tournier et al. 2008; Gaildrat et al. 2012; Di Giacomo et al. 2013; Mueller et al. 2015; Julien et al. 2016; Soukarieh et al. 2016; Tajnik et al. 2016). Such studies have inferred an unexpectedly large proportion of exonic sites to be involved in splicing (over 90% according to the highest estimate; Julien et al. 2016), suggesting that the need to maintain correct RNA processing could, on the contrary, be a major factor in CDS evolution. Are the results from these two independent fields of investigation comparable? Why do they appear to lead to such contrasting views on the prevalence and the evolutionary impact of exonic splice regulation (and of exonic RBP interactions more globally)? Finding answers to these questions will help us understand better the evolutionary dynamics of noncoding information within CDSs but might also shed light on other fundamental problems, such as estimating the extent to which variation in alternative splicing patterns is functional.

## Materials and Methods

### Caveats and Methodological Clarifications

The aim of the current work was to understand better how selection pressures related to RBP-binding have shaped human CDSs. It must be emphasized that our results are only indirectly relevant to the related problem of determining where on (pre-)mRNAs interactions with RBPs actually occur. Primary sequence is only one determinant of where an RBP binds, and can be more or less important depending on the protein (Li et al. 2014). For example, the binding preferences of many RBPs appear to be highly sensitive to local mRNA secondary structure (Wu et al. 2004; Aviv et al. 2006; Oberstrass et al. 2006; Skrisovska et al. 2007; Li et al. 2010; Masliah et al. 2013; Lambert et al. 2014). Because of this, our method, which consists solely in scanning the sequence for particular *k*-mers, cannot be used to determine individual binding sites with any accuracy. However, if the over-all density or rate of evolution of a set of motifs deviate from neutral expectations, this is likely an indication that selection has acted upon the motifs. It is precisely these kinds of patterns that we study and quantify in the current paper.

If one wishes to obtain a snapshot of the protein–RNA interactions occurring in a population of cells at a given time, approaches such as ours are inappropriate. One then typically turns to various genome-wide experimental methods based on the crosslinking and immunoprecipitation of protein–RNA complexes, followed by high-throughput sequencing of the RNA fragments (CLIP-seq) (Licatalosi et al. 2008; Xue et al. 2009; Hafner et al. 2010; Konig et al. 2011; Van Nostrand et al. 2016). Although caveats apply (Kishore et al. 2011; Sugimoto et al. 2012; Friedersdorf and Keene 2014; Lambert et al. 2014), these methods are the state of the art for localizing RBP target sites on RNA.

However, data from CLIP-seq studies cannot easily be used to assess the long-term evolutionary impact of RBP–protein interactions, which is our goal in this article. By its very nature, the method does not distinguish between spurious binding and evolutionarily relevant interactions − that a given interaction is observed, even if repeatably and significantly above background, does not always mean that it has fitness relevance or an impact on sequence evolution. In addition, CLIP-seq data does not allow one to precisely control for nucleotide composition biases, a crucial confound in any analysis of molecular evolution. Finally, producing estimates of global evolutionary impact is further rendered difficult by a high false negative rate (Darnell 2010; though see Van Nostrand et al. 2016).

Computational methods, such as the one used in this work, are therefore more appropriate for answering questions on sequence evolution. Several caveats must, nevertheless, be bourn in mind. Firstly, although the motifs used in this study were derived through experiments conducted on particular RBPs, there is nevertheless no direct link between motif and RBP during the sequence analysis. Similar motifs can be recognized by different RBPs (for instance, in our dataset, the motif *CCATACC* is associated to both poly(RC) binding protein 1 (PCBP1) and to heterogeneous nuclear ribonucleoprotein K (HNRNPK)). This means that when a set of motifs displays interesting distributional or evolutionary properties, there is no guarantee that this is necessarily due to interactions with the RBP to which we have associated that set of motifs, rather than to any other roles the motifs might have. We note that motif sets associated to RBPs that have been experimentally observed to preferentially bind within coding sequence are also at a greater density (raw and normalized) in coding regions than those predicted to bind elsewhere (see “Motif Sets That Are More Strongly Enriched Also Tend to Be More Conserved” section in the “Results” section). This suggests that the motif to RBP mapping does indeed have global validity. However, it is still advisable to limit interpretation to over-all patterns (such as the relationship between enrichment and conservation measures) rather than to draw conclusions regarding particular RBPs.

In addition, the extent of sequence-specificity is expected to vary between RBPs (Li et al. 2014; Jankowsky and Harris 2015). Therefore, if a set of motifs associated to a particular RBP distributes in accordance with random expectations, this does not necessarily mean that interactions with this protein are unimportant for CDSs. It may simply indicate that in vivo, sequence is not a very important determinant of where this RBP binds. On a similar note, the quality of the motif sets is likely to vary depending on the protein and the method used to derive the motifs, with different techniques plagued by different biases (Marchese et al. 2016). This could also partially explain why certain sets of motifs show stronger deviations from neutrality than others.

### General Methods

The majority of the analysis was conducted using custom Python 3.4.2. and Perl v5.22.2 scripts (code available at www.github.com/rosinaSav/RBP_motifs; last accessed January 27, 2017). Unless otherwise noted, only standard libraries, NumPy 1.9.1. (van der Walt et al. 2011) and Biopython 1.64 (Cock et al. 2009) were used. R version 3.2.1. (R Core Team 2013) was used for plotting and for pre-made statistical tests. Bedtools 2.19.1 (Quinlan and Hall 2010) was used for operations on sequence coordinates. The analysis of human and macaque was based on assemblies GRCh38 and MMUL1, with the annotations corresponding to Ensembl release 78 for CDSs and Ensembl release 85 for noncoding regions (Cunningham et al. 2015). For the mouse and rat analysis, genome assemblies GRCm38 and Rnor_6.0 with the annotations from Ensembl release 80 were used. The genome sequences were obtained from the UCSC database (Karolchik et al. 2004). Gene annotations were downloaded as .gtf files from the Ensembl FTP site (Cunningham et al. (2015); ftp.ensembl.org/pub, last accessed 25 August 2015 for human (release 78) and mouse, 30 October 2015 for rat and 19 August 2016 for human (release 85) and macaque). Ensembl BioMart was used for retrieving the macaque CDS sequences (Kinsella et al. 2011; http://www.ensembl.org/biomart/martview; last accessed 21 February 2015). The pairwise alignments of human and macaque noncoding regions were retrieved from the Ensembl Compara database (Herrero et al. 2016) using a local installation of the Ensembl database and API (release 85).

### The RBP Motif Sets

Consensus motifs for the various RBPs were retrieved from several sources, detailed below. Some sources store position weight matrices (PWMs) or position-specific scoring matrices (PSSMs), while others use consensus sequences. We converted the PWMs/PSSMs into consensus sequences by representing each site in the matrix as the IUPAC symbol corresponding to all those bases that presented a value greater than 0 (in the case of PWMs) or 0.25 (in the case of PSSMs) at that site.

#### RBPDB

The *all experiments* and *all proteins* CSV files were downloaded from rbpdb.ccbr.utoronto.ca/download.php (Cook et al. 2011; last accessed 11 November 2015). Those experiments that were not performed in *Homo sapiens*(*/Mus musculus*) or for *Homo sapiens*(*/Mus musculus*) RBPs, or that did not report a sequence motif were excluded. The consensus motifs from the remaining experiments were retained. In addition, PWMs were downloaded from the same website and converted into consensus sequences, as described above.

#### RBPmap

The RBPmap package was downloaded from rbpmap.technion.ac.il/download.html (Paz et al. 2014; last accessed 12 November 2015). PSSMs for human/mouse proteins were converted into consensus motifs. RBPmap does not distinguish between human and mouse and so the PSSMs retained for either analysis were identical, except that PSSMs originating from RNAcompete were ignored for mouse (this was to avoid including a large set of PSSMs determined originally for human in the mouse analysis).

#### SFmap

SFmap consensuses were obtained from sfmap.technion.ac.il/SF_list.html (Paz et al. 2010; last accessed 12 November 2015) and added to the list of motifs. SFmap does not distinguish between human and mouse and so all the motifs were included when analysing either species.

#### CISBP-RNA

The entire *Homo sapiens* dataset was retrieved from cisbp-rna.ccbr.utoronto.ca/bulk.php (Ray et al. 2013; last accessed 11 November 2015). The PSSMs labelled *direct* (signifying that the motifs were experimentally determined for that particular RBP rather than inferred from proteins with similar domains) were retained and converted into consensus sequences. Mouse consensuses were derived similarly, except that indirect PSSMs were also included.

Motifs from the different sources were then pooled. This resulted in 183 RBPs in human and 188 in mouse, each associated to a set of *k*-mers. *N* (fully ambiguous) bases at the very beginning or at the very end of motifs were removed. The motifs were then filtered to only leave those with length between 5 and 12 bases (included). Motifs that contained parentheses (signifying variable motif length) were removed. After this filtering step, 133 RBPs remained in human and 163 in mouse. However, because the source databases differed in naming conventions, some of the RBP identifiers that had been retained referred to the same protein. For human, the remaining RBP identifiers were therefore fed to Ensembl BioMart and converted to Ensembl gene identifiers. This step was undertaken to verify whether or not the identifiers were recognized as valid HGNC symbols. Those that were not were manually converted into HGNC symbols using the GeneCards database (www.genecards.org (Safran et al. 2010); last accessed 12 November 2015). For mouse, the protein identifiers were input into the Mouse Genome Informatics (MGI (Bult et al. 2016); last accessed 19 October 2016) web site as a batch query. The output was used to update all identifiers to the *current symbol* recognized for the protein. Hnrnpcl1, which was not recognized at all by MGI, was discarded. This step resulted in several synonymous identifiers being collapsed, leaving us with a total of 117 RBPs for human and 81 for mouse. Three of the human RBPs were removed from the dataset: microRNA 1236 (*MIR1236*; because it is a microRNA gene rather than an RBP), poly(A) binding protein, cytoplasmic 4 (PABPC4; the consensus was *AAAAAAA*, making normalization for dinucleotide composition impossible) and peptidylprolyl isomerase E (cyclophilin E) (PPIE; the consensus was *WWWWWW*, making it once again impossible to generate simulants). Pabpc4 was removed from the mouse set for similar reasons. The final number of RBPs retained was therefore 114 for human and 80 for mouse.

In human, two consensus sequences were added manually: the consensus *UUWGDUU* was added to ELAV like RNA binding protein 1 (ELAVL1), while the consensus *RWUUYAUUUWR* was added to ELAV like neuron-specific RNA binding protein 2 (ELAVL2). This is because in these cases, the retained motifs included both consensus sequences lifted directly from a database, as well as consensuses that we had derived from a PWM/PSSM. Both, however, were based on the same original publication. The new motifs were added to summarize these existing consensuses in a broader consensus that would combine the information from both sources.

For all RBPs, the remaining consensuses were then expanded into all the nonambiguous motifs that would match the consensus. Identical motifs were collapsed. This resulted in the final motif sets (additional file S1, Supplementary Material online).

### The Random Motifs (Used Only to Generate the Distribution Indicated by a Black Line in fig. 1*A*)

The sequence of the human genome (GRCh38) was obtained from the UCSC Genome Browser website (Karolchik et al. 2004). Only reference chromosomes were considered: unplaced, unlocalized and alternative sequences were excluded. The counts of each of the 4 DNA bases were summed across all the chromosomes and divided by the total number of canonical (*A*, *T*, *C* or *G*) bases.

In each of the RBP motif sets, the motifs were then replaced by random motifs of the same length. To generate a motif of length *k*, *k* canonical bases were randomly picked (using *numpy.random.choice()*), with the probability of each base being chosen corresponding to its mononucleotide frequency in the human genome, as determined above.

### The Sequence Sets

#### Full CDSs

The sets of intron-containing and intronless human CDS sequences were the same as those used in Savisaar and Hurst (2016). The methods used for generating these sequence sets were detailed in the cited publication and will only briefly be summarized here. All intronless/intron-containing ORFs from *GRCh38* were downloaded from the Ensembl database (release 78). For intronless genes, only ORFs from genes that exclusively produced intronless transcripts (according to the transcript annotations available in the Ensembl database) were kept. The ORFs were then checked for reading frame integrity and completeness. If several transcripts corresponded to one gene, the one with the longest ORF was kept. The remaining transcripts were then aligned to macaque orthologs. Only those that had an ortholog to which they aligned with a *d_S_* below 0.2 and a *d_N_*/*d_S_* below 0.5 were kept. This filtering step was necessary to minimize the proportion of pseudo-genes in the set. Finally, the sequences were BLASTed all against all and clustered into paralogous families based on the results. Mouse full CDSs were obtained similarly (using *GRCm38*, Ensembl release 80), except that the *d_S_* threshold was set to 0.3 during the filtering.

#### Exon Flanks and Cores

To generate the sets of exon flanks/cores, we recovered all of the internal fully coding exons in our set of human intron-containing genes that were at least 211 base pairs long (based on Ensembl release 78 annotations; only one randomly picked gene was considered per paralogous family). The exons were trimmed so as to both start and end with full codons. (The length threshold was set to 211 because three 69 base pair long nonoverlapping regions were to be extracted from each exon (3×69 = 207) and at least 4 base pairs had to be left over in case any nucleotides were lost because of trimming.) Three sequence regions were then extracted: the first 69 base pairs at the 5′ end of the exon, the final 69 base pairs at the 3′ end and 69 base pairs from the very centre. If the number of codons separating the 5′ region from the 3′ region was even, meaning that it was not possible to define the exact mid-point (when 69 is subtracted from an even number, the result is odd), the core was defined so as to be separated from the 5′ flank by *n* codons and from the 3′ flank by *n − 1* codons.

#### Noncoding Sequences

A set of human CDSs was retrieved and filtered for ORF integrity and conservation level as per the procedure used above, except that Ensembl release 85 annotations were used. The sequences were clustered into putative paralogous families as described above. The chromosomal coordinates of the introns, 5′-UTRs and 3′-UTRs associated to the transcripts in the set were retrieved based on Ensembl gene annotations (release 85, one transcript was randomly picked from each paralogous family). In addition, 100 base pairs were extracted from immediately upstream and immediately downstream of each exon (only the intronic flank was used for terminal exons). The full introns set was filtered further, firstly by randomly picking only one intron from each transcript (to limit the size of the dataset for computational reasons), and secondly by excluding all introns that overlapped with any exons, as defined by Ensembl annotations.

The coordinates were then used to retrieve the LASTZ_NET human-macaque pairwise alignment from a local installation of the Ensembl Compara database (release 85), using the Ensembl API. Only alignments that corresponded to a single genome alignment block and that contained no *N* bases in either the human or the macaque sequence were retained.

### Motif Density and ND

To calculate the density of the full set of motifs, we counted the number of bases that overlapped with any of the motifs in the set in each CDS and divided this count by the length of the CDS. We used the full CDS, that is to say, all of the coding sequence between the start and the stop codon in the relevant transcript variant. We did not take into consideration the positions of exon-exon junctions. Bases encompassed by more than one motif were only counted once (i.e., overlapping motifs were collapsed). We calculated an ND value separately for each gene (see main text for the calculation of ND and below for the generation of simulant motifs) and used the median density and the median ND as our statistics (averaged across paralogous families). Because less data was available, a different approach was used when calculating the densities of individual motif sets (the motifs associated to a particular RBP). Namely, rather than producing a density estimate per gene, we summed the number of overlapping bases across all the sequences and divided that by the summed length of the sequences. This produced a single point estimate for density and for ND for each set of motifs. Counts and lengths were averaged across paralogous families before the division step.

About 1,000 (for the full set density analysis in human CDSs) or 100 (for the full set density analyses in human noncoding sequences and for the mouse analysis) simulant versions of the RBP motifs set were generated in order to calculate the enrichment *P* value and ND. The motifs were divided into dinucleotides in the two possible phases. To generate each of the motifs in the 1,000/100 simulant sets, the necessary number of dinucleotides were sampled randomly with replacement from the pool of dinucleotides. If the motif length was odd, an additional base was sampled from the mononucleotide composition of the motif set. This resulted in 1,000/100 sets of simulant motifs, with the motif number, motif lengths and the dinucleotide composition matched to the true set of motifs (the match being approximate in the case of dinucleotide composition). The resulting simulants were screened, such that no simulated motifs were allowed that also appeared in the set of real motifs. In addition, no simulants could contain a mononucleotide run that was longer than the longest run of that base in the real motif set. Finally, all the motifs within a particular simulant set had to be unique. In the analysis of motif enrichment independent of stop codon content, simulants were additionally constrained to be devoid of the substrings *TAA*, *TGA* and *TAG* (see supplementary text 3 in additional file S5, Supplementary Material online). Simulants were generated similarly for the individual motif sets (1,000 simulant sets were always used).

Hits were then predicted in the sequences to each of the simulated motif sets, generating an empirical distribution of simulated density values. From this distribution, ND and *P* were derived as described in the main text (see above for differences between the processing of the full motifs set and the individual sets). The normalization step is even more important when considering individual sets of motifs (motifs grouped based on the putative cognate RBP), as in addition to controlling for nucleotide composition biases, this step largely eliminates the confounding factor of the sets varying in the number and length of the motifs. For instance, the smallest sets only consist of a single motif whereas the largest in human − composed of *k*-mers putatively recognized by the RBP transformer-2 protein homolog beta (TRA2B) − has 218 motifs.

After calculating the density of the individual motif sets, we noticed that some were very rare, leading to concerns over whether there was sufficient information to reliably estimate ND and other parameters in those cases. In human, we decided to only include those motif sets in the subsequent analysis that filled one of two criteria: either the hits to the real motifs totalled at least 100 bp in the intron-containing CDSs, as well as in each of four other sequence sets (intronless CDSs, exon 5′ flanks, exon cores and exon 3′ flanks) or the hits to at least half of the simulant sets did. The reasoning behind this rule was that if the real motifs were rare, whereas the simulants were not, or the other way around, then this was potentially a biologically meaningful pattern, whereas if both were rare, then one simply had a lack of information. The mouse filtering was similar, except that only the density in intron-containing CDSs was considered. Only one RBP was filtered out in this process (Raver1).

### Rate of Evolution at Synonymous Sites

*d_S_* estimates were calculated identically to Savisaar and Hurst (2016), which details the methods used. Only a brief summary will therefore be provided here. Sequence regions overlapping with RBP motifs were extracted and aligned to homologous regions in macaque (*Macaca mulatta*). The rate of evolution at synonymous sites was calculated using the Goldman and Yang (Goldman and Yang 1994) method, as implemented in the *codeml* programme that is part of the Phylogenetic Analysis by Maximum Likelihood (PAML) (Yang 2007) suite. This procedure was then repeated for each of 1,000 simulant sets, enabling us to calculate a normalized *d_S_* estimate and an enrichment *P* value. One randomly picked gene was considered from each paralogous family.

### Rate of Evolution of Noncoding Sequence

To calculate rates of evolution for noncoding sequences, the *baseml* programme from the PAML suite was used (*model* = 1). The statistic used, termed here *d_NC_*, corresponds to the tree length reported by the programme.

### Conservation at 4-Fold Degenerate Sites Overlapping Different Dinucleotides

The 4-fold degenerate sites in intron-containing sequences were divided into two groups: those that overlapped an RBP motif hit and those that did not. Within each class, we then further sub-divided the sites based on the overlapping dinucleotide. Each site was counted twice, once as belonging to the dinucleotide in which it was the second base and once as belonging to the dinucleotide in which it was the first base. For each dinucleotide class within either site type (motif or nonmotif), we determined the fraction of sites where the orthologous position in macaque did not exhibit the same base as in human. In order to obtain an over-all estimate of the difference in evolutionary rate between motif and nonmotif, we averaged the rates calculated for different dinucleotides but weighted them by the frequency of each dinucleotide within the subset of sites overlapping with RBP motifs, thereby controlling for any differences in dinucleotide composition between motif and nonmotif regions. A random member was included fro each paralogous family.

### Human-Macaque Comparison at 4-Fold Degenerate Sites That Are a Single Base Substitution Away from a Putatively Avoided Motif in Human

We determined all 4-fold degenerate sites in our set of full human intron-containing CDSs (one randomly picked gene from each paralogous family) such that a single base substitution at that site could generate a putatively avoided motif (a motif with enrichment *P* value above 0.9 in full intron-containing CDSs). We then scored each site based on the identity of the orthologous macaque base. The following scores were possible: 0 (the base present in macaque is either identical to that present in human or is a base other than the one(s) that would give rise to an avoided motif in human), 0.25 (the base present in macaque would give rise to an avoided motif in human. Of the 3 possible base substitutions in human, all three would generate a putatively avoided motif), 0.5 (the base present in macaque would give rise to an avoided motif in human. Of the three possible base substitutions in human, two would generate a putatively avoided motif) and 0.75 (the base present in macaque would give rise to an avoided motif in human. Of the three possible base substitutions in human, only the one used in macaque would generate a putatively avoided motif). The scores were summed across all sites and the sum divided by the number of sites considered. The analysis was then repeated on 1,000 sets of simulated motifs that broadly matched the dinucleotide composition of the putatively avoided motifs, allowing us to calculate a *P* value for the statistic obtained.

The reasoning behind the scoring system is that macaque presenting the base that would generate the avoided motif in human constitutes stronger evidence against avoidance against that motif if other substitutions were possible that would not have generated the motif than when any substitution would have led to a putatively avoided motif. Note that there are several caveats to this analysis. Firstly, because we did not use an outgroup, we do not know on which the branch the substitution occurred in cases where the human and the macaque sequence differ. However, more frequently than expected by chance, macaque also does not have the base that would give rise to the putatively avoided motif in human, suggesting that this was also the case in the most recent common ancestor. Secondly, it is possible that a substitution that would generate a particular putatively avoided motif would simultaneously disrupt another such motif that overlaps with the first, meaning that the substitution would not necessarily lead to an increase in avoided motif density. Our analysis did not consider this issue. Thirdly, in macaque, we only analysed the base present at the particular site considered. We therefore did not account for any other potential differences between human and macaque at sites nearby, which could mean that even though a particular substitution would lead to a putatively avoided motif in human, it might not do so in macaque.

### Annotating the Motif Sets Based on the Properties of the Associated RBP

To annotate the motif sets based on the binding profile of the associated RBP, we searched the literature for high-throughput crosslinking and immunoprecipitation (CLIP-seq) studies conducted on that RBP. Only one study was considered per RBP. Each RBP was annotated as either *CDS* or *other* based on whether or not the study reported an enrichment of binding clusters in the CDS (if no CLIP-seq studies could be found, the RBP was annotated as *NA*). The interpretation of the authors was followed when deciding how to report the results of a particular study. For instance, if the authors reported CDS clusters to be rare but did not control for the fact that the combined length of coding regions is much shorter than that of introns, we still annotated the RBP as *other*. The annotations, as well as the sources used, are listed in supplementary spread sheet 5 in additional file S4, Supplementary Material online.

### Expression Analysis

The phase 1 and 2 combined normalized .osc file was retrieved from the FANTOM5 website (http://fantom.gsc.riken.jp/5/datafiles (Fantom Consortium et al. 2014); last accessed 11 February 2016). The data was filtered to only leave samples where the sample name contained the substring *adult*, *pool1*. All brain tissues except for the full brain sample and the retinal sample were removed. Peak coordinates were converted to *hg38* coordinates using CrossMap 0.2.2. (Zhao et al. 2014). For each transcript in our set of intron-containing protein-coding genes (based on Ensembl release 78), we defined a region of 1001 base pairs centered on the start coordinate of the Ensembl transcript annotation as the promoter and associated all peaks that overlapped that promoter to that peak. If several peaks were associated to a single transcript, we summed the tags per million (TPM) within each sample across the peaks. The TPM were then averaged across paralogous families.

## Supplementary Material

Supplementary data are available at *Molecular Biology and Evolution* online.

## Supplementary Material

Supplementary Data
