# Supplementary material for: Both Maintenance and Avoidance of RNA-Binding Protein Interactions Constrain Coding Sequence Evolution
Source: Mol Biol Evol. 2017 Jan 30;34(5):1110–26. doi: 10.1093/molbev/msx061 (PMC5400389; doi:10.1093/molbev/msx061)
Supplement: Supplementary Data [file msx061_Supp.zip › Additional_File_5.pdf]

Supplementary Material

Additional Files

Additional File 1: The RBP motifs used in human and mouse. The human data also has information on whether the motif set passed the information content threshold for inclusion in the analysis of individual motif sets.

Additional File 2: Putative non-singleton paralogous families of human intron-containing CDSs (based on Ensembl relese 78). Putative non-singleton paralogous families of mouse intron-containing CDSs.

Additional File 3: Putative non-singleton paralogous families of human intronless CDSs (based on Ensembl release 78).

Additional File 4: The supplementary spreadsheets.

Additional File 5: This file. Overview of the additional files and spread sheets. Supplementary Texts. Supplementary Figures.

Supplementary Spread Sheets

Supplementary Spread Sheet 1: Density parameters of RBP motifs in human intron-containing CDSs (one data point per gene).

Supplementary Spread Sheet 2: Density parameters of RBP motifs in human intron-containing CDSs (one data point per RBP).

Supplementary Spread Sheet 3: Density parameters of random motifs in human intron-containing CDSs (one data point per RBP).

Supplementary Spread Sheet 4: Conservation parameters of RBP motifs in human intron-containing CDSs (one data point per RBP).

Supplementary Spread Sheet 5: The experimentally determined binding preferences of RBPs.

Supplementary Spread Sheet 6: Results from the analysis of human-macaque divergence at human fourfold degenerate sites that are a single base substitution away from a putatively avoided motif (one data point per RBP).

Supplementary Spread Sheet 7: Density parameters of RBP motifs in human intronless CDSs (one data point per gene).

Supplementary Spread Sheet 8: Density parameters of RBP motifs in human intronless CDSs (one data point per RBP).

Supplementary Spread Sheet 9: Conservation parameters of RBP motifs in human intronless CDSs (one data point per RBP).

Supplementary Spread Sheet 10: Density parameters of RBP motifs in mouse intron-containing CDSs (one data point per gene).

Supplementary Spread Sheet 11: Density parameters of RBP motifs in mouse intron-containing CDSs (one data point per RBP).

Supplementary Spread Sheet 12: Conservation parameters of RBP motifs in mouse intron-containing CDSs (one data point per RBP).

Supplementary Spread Sheet 13: Density parameters of RBP motifs in human 5'UTRs (one data point per sequence).

Supplementary Spread Sheet 14: Density parameters of RBP motifs in human 3'UTRs (one data point per sequence).

Supplementary Spread Sheet 15: Density parameters of RBP motifs in human introns (one data point per sequence).

Supplementary Spread Sheet 16: Density parameters of RBP motifs in human intronic regions immediately upstream from an exon (one data point per sequence).

Supplementary Spread Sheet 17: Density parameters of RBP motifs in human intronic regions immediately downstream from an exon (one data point per sequence).

Supplementary Spread Sheet 18: Expression parameters of human intron-containing genes.

Supplementary Spread Sheet 19: The sizes of individual RBP motif sets in both human and mouse.

Supplementary Texts

Supplementary Text 1: Estimating RBP motif conservation separately for each dinucleotide.

The RBP motif conservation reported in section 1.2. of *Results* is highly dependent on the success of our procedure for controlling for dinucleotide content. If certain highly mutable dinucleotides (such as *CG*) were systematically over-represented in simulants when compared to the true motifs, then this could artifactually cause RBP motifs to appear slow-evolving. To ascertain whether the decrease in evolutionary rates in RBP motifs was a systematic effect that concerned more or less all dinucleotides, rather than simply a reflection of the over-representation of certain highly unstable dinucleotides in the simulants, we assessed conservation separately for each dinucleotide. We divided all the fourfold degenerate sites in our CDSs into two classes (using one randomly picked gene from each paralogous family) – those that overlapped with hits to RBP motifs and those that did not. Within either category, we then further grouped the sites based on the dinucleotide that they were part of (counting each site twice, once for the dinucleotide in which it was the second base and once for the dinucleotide in which it was the first base). Finally, for each such subgroup of sites, we asked how frequently the base observed at the orthologous position in macaque was different from that present in human, thereby obtaining an estimate for the rate of evolution at fourfold degenerate sites (d_4) for each dinucleotide at both motif sites and non-motif sites. We found that for the majority of dinucleotides (12/16), d_4 was lower at sites overlapping RBP motifs than at other sites. This proportion is significantly greater than expected by chance ($\chi^2 \approx 4$, $p < 0.05$). We also obtain a significant result when we compare the values using a paired one-tailed Wilcoxon signed rank test ($p \approx 0.017$). These results indicate that the reduction in evolutionary rates is unlikely to be driven solely by a few unusual dinucleotides that would be both over-represented in simulants when compared to true motifs and particularly fast-evolving.

To obtain an estimate for the global reduction in d_4 in RBP motifs, we can average the rates obtained across all dinucleotides at either motif or non-motif sites, weighting each dinucleotide by its frequency at motif sites (thereby controlling for differences in dinucleotide frequencies between motif and non-motif). This produces an average d_4 of ≈0.0087 for motif sites and of ≈0.0089 for the rest of the sequence, a difference of about 1.78%. This is a weaker effect than that obtained when considering d_S rates. This indicates that the decrease in d_S in RBP motifs might indeed partially be due to imperfections in the process of generating simulants, although note that our method of calculating d_4 lacks the sophistication of the Goldman and Yang (1994) method used for d_S, which may also explain part of the discrepancy (for example, we apply no correction for multiple hits). Note also that the effect is locally stronger in exonic sub-regions that can be hypothesized to be particularly relevant for splicing (a decrease of *ca.* 4.62% is observed when only the 5' flanks of exons are considered).

Supplementary Text 2: RBP-related constraints appear no weaker in intronless than in intron-containing genes, underlining the importance of splicing-independent factors in shaping RBP motif usage.

We hypothesized that because intron-containing genes would have a greater need to interact with splice factors than intronless ones, their evolution would be under stronger RBP-related constraints. We therefore assembled a set of 344 intronless genes and clustered them into paralogous families, resulting in 157 data points (Supplementary File 3). We then proceeded to calculate the density and synonymous rate of evolution of RBP motifs also in the CDSs of these genes (Supplementary Spread Sheet 7). Contrary to our expectations, we found both enrichment and conservation to be *stronger* in intronless than in intron-containing CDSs (Table 1), and the over-all reduction in d_S across full sequences to round up to ≈3.3%. There is therefore no evidence for weaker RBP-related constraints in genes that lack introns. This finding might suggest that a sizable fraction of the functional interactions occurring between CDSs and RBPs are relevant to processes other than splicing.

	intronless CDSs	intron-containing CDSs
raw motif density	≈0.571	≈0.573
ND	≈0.153	≈0.115
enrichment p	≈0.001	≈0.001
raw d_S	≈0.065	≈0.064
normalized d_S	≈−0.058	≈−0.041
conservation p	≈0.001	≈0.003
over-all reduction in d_S	≈−0.033	≈−0.024

Table 1: Statistics of motif density and conservation in intronless and intron-containing genes (one data point per gene).

	intronless CDSs	intron-containing CDSs	significance of the difference from a paired two-tailed Wilcoxon signed rank test	Spearman rank correlation between intronless and intron-containing
median raw density	≈0.003	≈0.003	≈0.343	$\rho \approx 0.931$, $p < 2.2 * 10^{-16}$
median ND	≈−0.077	≈−0.008	≈0.678	$\rho \approx 0.833$, $p < 2.2 * 10^{-16}$
median raw d_S	≈0.068	≈0.062	≈0.105	$\rho \approx 0.506$, $p \approx 1.406 * 10^{-6}$
median normalized d_S	≈0.021	≈−0.032	≈0.880	$\rho \approx 0.454$, $p \approx 2.069 * 10^{-5}$

Table 2: Statistics of motif density and conservation in intronless and intron-containing genes (one data point per RBP motif set).

In addition to calculating the over-all constraint, we can also compare the distributions of enrichment and conservation estimates obtained by predicting hits to individual RBP motif sets grouped based on the RBP predicted to recognize them (Supplementary Spread Sheets 8 and 9). There are no significant differences between intronless and intron-containing sequences with regards to any of the parameters of density or of evolutionary rate considered in our analysis, and all of these statistics are significantly correlated between intronless and intron-containing sequences (Table 2). Moreover, of the 81 motif sets analysed, about equal numbers showed greater ND in intron-containing than in intronless CDSs (44 motif sets), and the other way around (37) ($\chi^2 \approx 0.605$, $p > 0.95$). Similarly, there are 43 sets that have lower normalized d_S in intron-containing CDSs and 38 that show the opposite pattern ($\chi^2 \approx 0.389$, $p > 0.95$). The data from individual motif sets therefore also suggests that RBP motifs show no tendency to be either less strongly enriched or less conserved in intronless CDSs than in intron-containing ones.

In conclusion, RBP-related constraints appear just as relevant to intronless as they are to intron-containing CDSs. We note that this effect is unlikely to be due to the presence of retrotransposed duplicates of intron-containing genes in our dataset, as intronless CDSs mimic intron-containing ones not only in terms of their patterns of *k*-mer enrichment but also with regards to *k*-mer conservation, suggesting that the two types of sequences are under broadly similar functional constraints. This might imply that splicing-independent factors play an important role in shaping the RBP motif patterns within CDSs.

Supplementary Text 3: Could differences in stop codon content between motif sets explain some of the variation in ND?

If certain motifs contain the trinucleotide *TGA*, *TAA* or *TAG*, then it would be expected that they would be less frequent than simulants in coding sequences, simply because of the definitional lack of in-frame stop codons within coding regions. Because the simulant motifs were generated based on the dinucleotide rather than the trinucleotide content, they might not contain the stops present in the real motifs and would therefore have a higher frequency, leading to low ND. Similarly, if other motif sets have a high AT content but do not contain stops, this might inflate our estimate of enrichment as stops might easily occur in the simulants. Could this factor explain some of the variation in ND and, potentially, the extreme distribution of p-values?

To test this hypothesis, we classed the motif sets into two groups based on whether or not any of the motifs in the set contained a stop. The two classes of motif sets presented similar raw density values in intron-containing CDSs ($p \approx 0.542$, one-tailed Mann-Whitney U-test), however, the stop-containing sets exhibited lower ND (median 0.021 *vs* −0.092), a difference that is nearly significant ($p \approx 0.068$, one-tailed Mann-Whitney U-test, Supplementary Figure 2 A). The possibility that differences in stop codon content could be contributing to the enrichment/depletion patterns observed therefore cannot be excluded. This can either be a trivial methodological observation or it could be telling us something interesting about the evolution of RBP sequence specificities, namely that those proteins that function by binding within coding sequences have been selected to recognize motifs that do not contain stops. If the latter is true, then the differences in ND should be primary to the stop content, that is to say, the stop-containing sets should have lower ND regardless of the fact that they contain stops.

We therefore again scanned intron-containing protein-coding genes but first removed all those motifs that contained a stop codon. We also constrained the process of generating simulants so as not to allow simulant motifs that contained stops, thereby removing all effect of stop codon content. We then compared ND in those motif sets that had previously contained stops to those that had not. There was no significant difference ($p \approx 0.258$, one-tailed Mann-Whitney U-test; Supplementary Figure 2 B), suggesting that the near-significant effect detected before the removal of stops was primarily methodological. Importantly, however, even after the removal of stops from both the real motifs and the simulants, the tendency for extreme p-values remained (Supplementary Figure 3): the ratio of p-values below 0.1 or above 0.9 to those less extreme was still significantly greater than in random motifs ($\chi^2 \approx 124.981$, $p < 0.001$; see main text for further details). The same was true for the ratio of p-values above 0.9 to all others ($\chi^2 \approx 295.530$, $p < 0.001$). Differences in stop codon content between motifs and simulants may therefore contribute somewhat to determining ND values but do not explain the over-all pattern.

Supplementary Figures

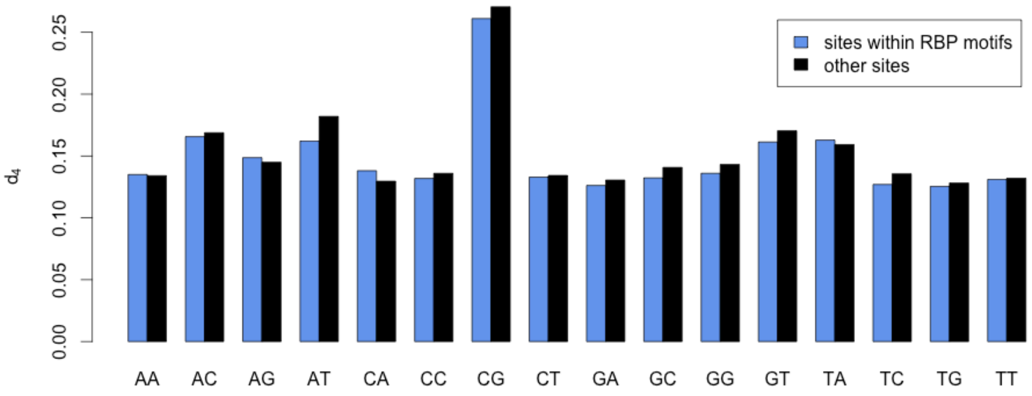

Supplementary Figure 1: d_4 of different dinucleotides at sites that either do (blue) or do not (black) overlap RBP motifs.

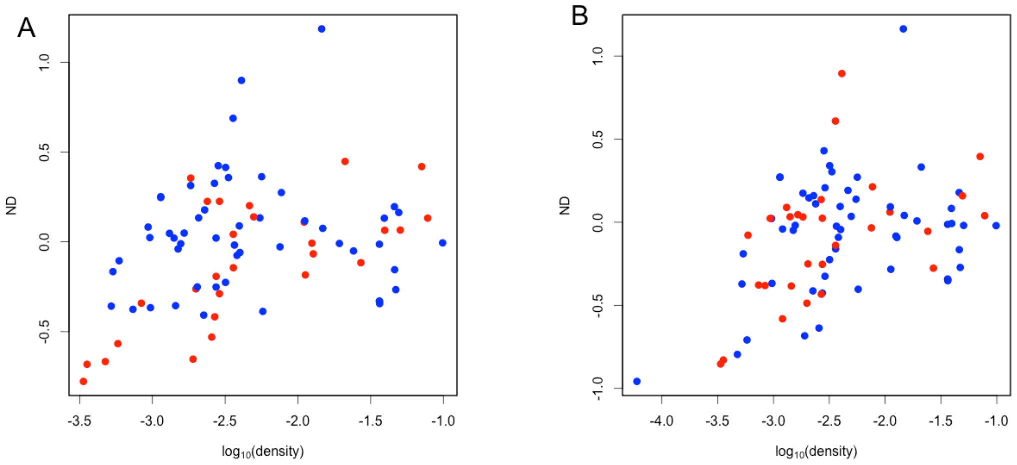

Supplementary Figure 2: A. The raw and normalized densities (ND) of RBP motifs grouped by the RBP predicted to recognize them. Red: at least one of the motifs in the set contains a stop codon. Blue: none of the motifs in the set contain a stop codon. B. As A., except that motifs that contain stops have been removed and no stops were allowed in simulant motifs. The colour-coding is based on whether the original motif sets contained a stop or not (that is to say, it is based on identical data to the colour-coding in A).

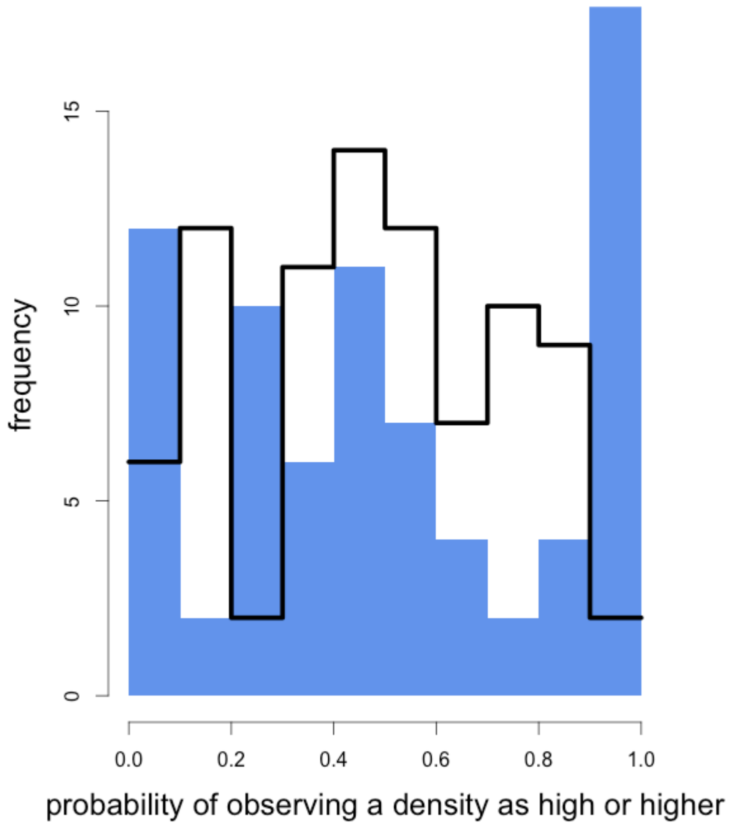

Supplementary Figure 3: As Figure 1A in the main text but with all motifs that contained a stop codon removed and without allowing stops in the simulants.

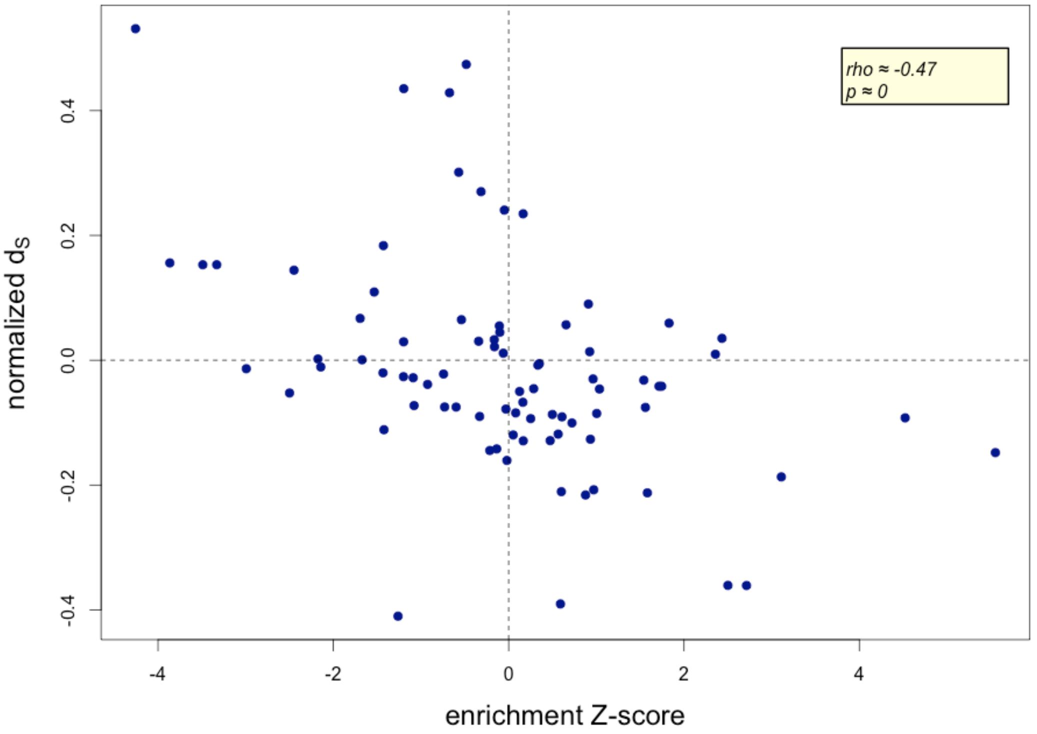

Supplementary Figure 4: the correlation between a motif set's normalized d_S and its enrichment *Z*-score. The *Z*-scores were calculated by subtracting the true density from the mean simulated density and dividing the difference by the standard deviation of simulated densities.

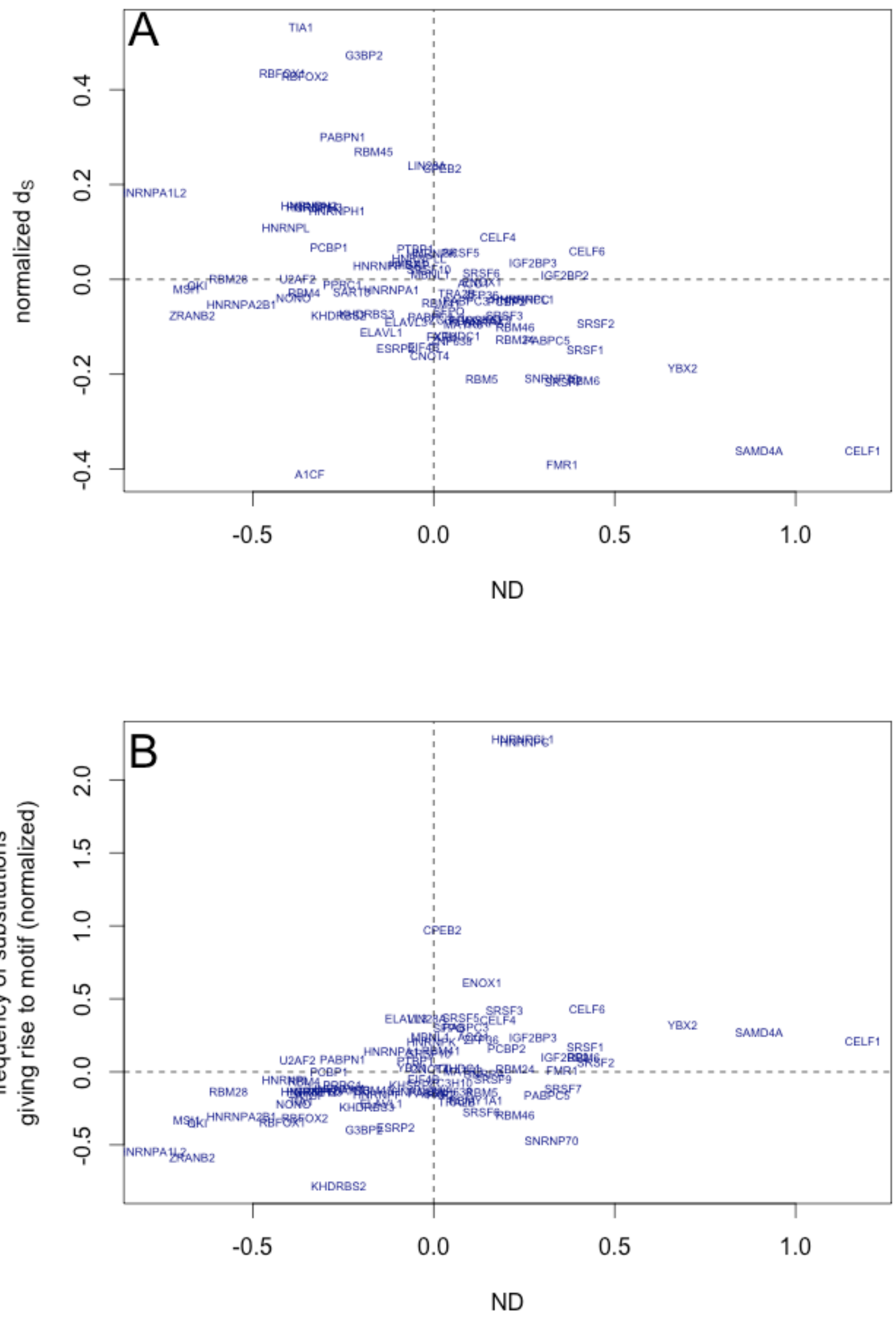

Supplementary Figure 5: As Figure 2A in the main text, but with each data point labelled with the HGNC symbol of the gene encoding for the protein that is predicted to recognize the motifs.

References

Goldman N, Yang Z. 1994. A Codon-based Model of Nucleotide Substitution for Protein-coding DNA Sequences. Mol Biol Evol 11:725-736.
